# Mining the Dynamic Genome: A Method for Identifying Multiple Disease Signatures Using Quantitative RNA Expression Analysis of a Single Blood Sample

**DOI:** 10.3390/microarrays4040671

**Published:** 2015-12-10

**Authors:** Samuel Chao, Changming Cheng, Choong-Chin Liew

**Affiliations:** 1 GeneNews Ltd., 445 Apple Creek Blvd. Unit 220, Markham, ON L3R 9X7, Canada; 2 Shanghai Biomedical Laboratory, Shanghai 200436, China; E-Mail: changming_cheng@shbiochip.com; 3 GeneNews Diagnostics Sdn Bhd, 213 Macalister Road, Georgetown, 10400 Penang, Malaysia

**Keywords:** blood transcriptomics, genomics, microarray, methodology for data analysis, diagnostics

## Abstract

Background: Blood has advantages over tissue samples as a diagnostic tool, and blood mRNA transcriptomics is an exciting research field. To realize the full potential of blood transcriptomic investigations requires improved methods for gene expression measurement and data interpretation able to detect biological signatures within the “noisy” variability of whole blood. Methods: We demonstrate collection tube bias compensation during the process of identifying a liver cancer-specific gene signature. The candidate probe set list of liver cancer was filtered, based on previous repeatability performance obtained from technical replicates. We built a prediction model using differential pairs to reduce the impact of confounding factors. We compared prediction performance on an independent test set against prediction on an alternative model derived by Weka. The method was applied to an independent set of 157 blood samples collected in PAXgene tubes. Results: The model discriminated liver cancer equally well in both EDTA and PAXgene collected samples, whereas the Weka-derived model (using default settings) was not able to compensate for collection tube bias. Cross-validation results show our procedure predicted membership of each sample within the disease groups and healthy controls. Conclusion: Our versatile method for blood transcriptomic investigation overcomes several limitations hampering research in blood-based gene tests.

## 1. Introduction

### Somatic Versus Dynamic Genome

The human genome can be explored in two different dimensions: the somatic genome and the dynamic genome. The somatic genome is the heritable DNA structure of an organism, with mutational heterogeneity that can be either the cause or effect of disease. To investigate hereditable factors and somatic mutations in disease, researchers have explored the somatic genome, using such methods as DNA sequencing technologies, single nucleotide polymorphism arrays, and genome-wide association studies. The results have been biologically informative and have produced a few clear medical and clinical successes, such as BRCA and HER2 testing in breast cancer. However, in general, results have been disappointing [1]. A fundamental problem with cancer DNA genome studies is that the genetic mutations for any given cancer type discovered by modern sensitive analytical methods number in the hundreds, and the determination of the true somatic mutation(s) driving cancer progression is difficult in the clinical setting for any individual patient [2]. Furthermore, cancer therapies targeted at a single somatic mutation have proven to have limited effect over time, because of resistance caused by cellular somatic evolution [3]. As human cancer is usually polypoid, containing subpopulations of multiple aneuploid cancer tumor cells, aneuploidy is also the hallmark of cancer cells in general [4]. Ploidy sequencing has been proved to be beneficial in revealing the somatic evolution of cancer tumor cells [5]. However, the clinic application of DNA ploidy assessment is very limited for cancer detection because it is still controversial that aneuploidy is the cause of cancer [6].

The other approach—exploring the dynamic genome—is one that we believe to be more powerful for clinical applications. This approach investigates not the DNA of an organism, but the transcriptional activity of an organism’s genes. The result of this activity is the transcriptome: the complete set of RNA transcripts present in a cell or tissue at any one time. Although the DNA of a particular cell or tissue, the genome, is uniform throughout the organism and except for infrequent random mutations, essentially unchanging, its transcriptome may vary according to the current physiological status of the cell, tissue or organism. Since mRNA profiles will alter in response to the cellular environment, the transcriptome will always be changing in response to immune factors, drugs, disease onset and progression, and healing [7].

To date, the dynamic genome has best been interrogated using microarray studies. Microarray chips can provide a snapshot of an organism’s gene expression activity at a given time. Compared to RNA sequencing, microarray is more established and cost-effective in analyzing the expression of defined genes by high throughput methods. Furthermore, microarray data is not as complex as that of RNA sequencing, which make it easier to analyze and apply widely to various fields. However, traditional, tissue-based microarray studies have a number of disadvantages. Invasive biopsies can be obtained only in very late stage disease, at transplant or after death in the case of difficult-to-access organs such as lung, breast, prostate, heart, and brain. For these reasons, tissue-based microarray is less useful for research in early-stage disease, or in the clinic.

One additional limiting factor in any tissue-based technology is the problem of heterogeneity. Diseased cells are not necessarily homogeneously distributed throughout tissue, and in cancer, malignant cells can differ from each other in their mutations [8]. Thus, analysis based on solid biopsy needs to take these factors into account by taking multiple invasive samples. However, this requirement increases the cost of the test and the test’s inconvenience to patients. To avoid this problem, so called “liquid biopsies” attempt to detect circulating tumor cells from a blood sample. While the presence of circulating tumor cells is a strong prognostic factor for overall survival in certain cancer patients, the clinical significance of circulating tumor cells in most patients is still unknown [9]. Furthermore since circulating tumor cells are very few in number in early stage cancer, analysis requires extreme analytical sensitivity to detect what very few cells are present and at a cost of many false positives, making testing unreliable.

By contrast, white blood cells in peripheral blood provide a near ideal diagnostic sample. Blood sampling is a long-established and well-accepted procedure for disease diagnosis and monitoring. Whole blood is easy to access, and patients and physicians are accustomed to blood sampling. White blood cells are much more abundant than circulating tumor cells, which eliminates the challenge of analytical sensitivity. Furthermore, because the sample is liquid, the distribution of cells is homogeneous. This reduces or eliminates the need for multiple samples. Another advantage of using whole blood is that the immune cells in blood are biologically affected by disease located elsewhere in the body regardless of the tissue affected [10,11]. Thus the requirement for direct biopsy is reduced and even potentially eliminated. For these reasons blood-based transcriptomics has significant advantages over tissue-based biopsy technology. We propose this concept as the Sentinel Principle^®^, which employs the circulating blood cells as “sentinels” for detecting and responding to micro-environmental changes in the body.

Blood-based transcriptomics may also come to play an important role in detecting disease at an early stage in which clinically apparent pathological variants have not yet emerged. Blood cells act as transporter cells and as mediators of the immune response and are involved in the pathogenesis of many diseases [12]. Thus when physiological or pathological insults occur anywhere in the organism, the gene expression profile of the peripheral blood cells will change in order to carry and to transfer information to engage the immune system and maintain physiological homeostasis [13]. Our previous research has shown that peripheral blood cells respond differently to various pathological changes and thus analysis of differential gene expression can distinguish between and among diseases [14]. Furthermore, since the interaction between the immune system and disease usually precedes the occurrence of clinically pathological variation, the study of blood cell profiles might make it possible to detect diseases at an early stage. For instance, according to the cancer immunoediting theory, cancer has a long equilibrium phase in which tumor cells survive immune elimination and maintain a state of functional tumor dormancy [15]. During the equilibrium phase, there is no clinically detectable pathological variation, but the interaction of the immune system against cancer cells occurs covertly in the body. Our hypothesis is that early stage cancer and other diseases can be detected by analysis of variation in the gene expression profiles of peripheral blood.

Although blood-based transcriptomics research has great potential in clinical application, it has its own set of limitations. In order optimally to measure the expression levels of messenger RNA in whole blood, several challenges need to be overcome. First, whole blood introduces into sampling the factor of white blood cell population distribution. Second, the dynamic composition of blood cells in response to a constantly changing environment means that even copy numbers normalized to total cell count and sample volume may exhibit variability. Third, sampling technology can introduce additional artefacts, such as differences between EDTA and PAXgene™ (PreAnalytiX) collection tubes and protocols. Moreover, microarrays from different manufacturers each have their own peculiarities and need corrective measures tailored for each. These factors make the analysis of the data more complex and time consuming. A practical solution is required that can identify well-performing gene panels in the face of these challenges.

The most common of these limitations, the interference derived from different blood sampling technologies, cannot be ignored in blood-based transcriptomics research. The conventional method for drawing blood uses EDTA collection tubes, which inhibit clotting but do not stabilise intracellular RNA. When EDTA blood collection tubes are used, intracellular RNA needs to be isolated within four hours of collection, as RNA degrades rapidly. Therefore, EDTA collection tubes are not practical for clinical applications when tests involve RNA and the collection sites are far from the laboratory. To overcome this problem, PAXgene tubes contain reagents that stabilise RNA, allowing easy blood collection, storage and transport of blood samples [16]. However, excessive globin mRNA levels interfere with transcript measurement and increase variability. This difficulty is addressed by the use of specifically designed reagents with different degree of globin signal suppression. Thus, gene expression profiles derived from EDTA and PAXgene blood collection tubes are not completely consistent between samples drawn from the same patient and processed under different protocols, an inconsistency that may lead to confusing or contradictory results.

## 2. Experimental Section

In an earlier study we published the predictive performance of gene panels identified by the method developed by our group using a data set consisting of 631 blood samples collected in EDTA tubes and discriminating for three diseases with an area under the receiving operator characteristic curve (AUROC) ranging from 89% to 93% [17]. We later expanded the set to include 17 diseases represented by more than 1700 samples, and we were able to obtain similar prediction performance. However, because our samples were collected using EDTA tubes, the results would not be useful for many clinical applications, as discussed above.

We have therefore transitioned to PAXgene tube collection and have started to rebuild our disease panels. This offered us an opportunity to evaluate the performance of our method against an established statistical package, Weka [18], for a study in which samples were collected initially using EDTA tubes and then PAXgene tubes.

In this two-part study, we first present details of our method and demonstrate its ability to overcome a known and documented bias between samples collected in EDTA and PAXgene tubes. The PAXgene samples were processed using Nugen reagent kit (NuGen, San Carlos, CA, USA). By extension, this method should be able to overcome other biases, which may not be known in advance. Then, we present the results of this method applied to a new cohort of samples representing ten cancers and diseases collected in PAXgene tubes and processed using the current 3′ IVT PLUS reagent kit (Affymetrix, Santa Clara, CA, USA). We switched from the Nugen kit because the new reagent kit has better repeatability performance in our laboratories.

### 2.1. Demonstration of EDTA and PAXgene Collection Tube Bias Suppression

For the initial part of the study, we used blood samples that we collected for a liver cancer (hepatocellular cancer) study. The samples comprised blood taken from hepatocellular cancer (HCC) patients, chronic Hepatitis B (HpB) patients and healthy controls, all recruited in Malaysia under approved protocols [19]. All subjects gave their informed consent for inclusion before they participated in the study. The study was conducted in accordance with the Declaration of Helsinki. Our training set consisted of HCC patients: 26 HCC collected in PAXgene tubes and 20 HCC collected in EDTA tubes. We also had 28 blood samples taken from patients with chronic hepatitis B (HpB) infection collected in PAXgene tubes, 28 confirmed HCC-negative (control) samples also in PAXgene tubes and seven control samples collected in EDTA tubes. In addition, 830 samples from other studies (“other”) were collected in EDTA tubes. These “other” samples were assumed to be negative for HCC because it is a low-prevalence disease. The test set consisted of independent samples: 25 HCC collected in PAXgene tubes, 15 HCC collected in EDTA tubes, 27 HpB collected in PAXgene tubes, 27 controls collected in PAXgene tubes, 7 controls collected in EDTA tubes and 860 “other” samples collected in EDTA tubes (Table 1).

**Table 1:** Breakdown of samples for collection tube bias demonstration.

Group	Training Set			Independent Test Set		
	Paxgene	EDTA	SubTotal	Paxgene	EDTA	SubTotal
HCC	26	20	46	25	15	40
HpB	28	0	28	27	0	27
Control	28	7	35	27	7	34
Other	0	830	830	0	860	860
Total	82	857	939	79	882	961

### 2.2. New Samples and Cross-Validation

After we demonstrated that this method works well in separating the three groups of liver cancer study samples (HCC, HpB, control), we proceeded to apply the same method to a new set of 157 samples representing ten diseases and healthy controls collected and processed in Penang and Shanghai (Table 2).

**Table 2:** New samples for cross-validation.

Disease	PAXgene	Source
Lung Cancer	12	Malaysia
Liver Cancer	8	Malaysia
Nasopharyngeal Cancer	20	Malaysia
Prostate Cancer	25	Malaysia
Breast Cancer	12	Malaysia
Cervical Cancer	9	Malaysia
Colorectal Cancer	10	Malaysia
Ulcerative Colitis	10	China
Crohn’s Disease	9	China
Osteoarthritis	8	Malaysia
Healthy Controls	34	China/Malaysia
Total	157	85% Malaysia/15% China

### 2.3. Methods

#### 2.3.1. Blood Collection, RNA Isolation and RNA Quality Control

Peripheral whole blood was collected from patients in EDTA Vacutainer tubes (Becton Dickinson, Franklin Lakes, NJ, USA) and PAXgene tubes (PreAnalytix, Hombrechtikon, Switzerland). Whole blood RNA was isolated as described previously [20]. Isolated RNA was checked by using 2100 Bioanalyzer RNA 6000 Nano Chips (Agilent Technologies, Santa Clara, CA, USA). Samples were excluded from microarray analysis that did not meet the following quality criteria: RIN ≥ 7.0; 28S:18S rRNA ≥ 1.0. RNA quantity was determined by absorbance at 260 nm in a DU800 Spectrophotometer (Beckman Coulter, Brea, CA, USA) or a NanoDrop 2000c UV-Vis Spectrophotometer (Thermo Scientific, Wilmington, DE, USA).

#### 2.3.2. Microarray Hybridization and Probe Set Quality

We used the Affymetrix GeneChip Human Genome U133 Plus 2.0 microarray (Affymetrix) and the FDA-cleared, CE-IVD marked Affymetrix Gene Profiling Array cGMP U133 P2 microarray (Affymetrix) for this study. Expression data was extracted by using the MAS5 analysis method, as each microarray needs to be evaluated independently. This technique is directed towards allowing single sample predictions, which are more practical in a clinical setting.

We followed the MAQC list for the Affymetrix GenChip Human Genome U133 Plus 2.0 microarray and ignored probe sets that are not included in the list of MAQC for Affymetrix microarrays [21].

We then conducted an experiment to identify probe sets which exhibit stable results across 7 different chip lots using 4 replicates each, for a total of 28 hybridizations on a pooled mRNA sample extracted from whole blood (Table 3). Probe sets were ranked according to observed variability across the 28 hybridizations.

**Table 3:** Four replicates across seven microarray lots.

Sample ID	Lot #	RAW Q	Background	SF	Present	GAPDH 3′/5′	Actin 3′/5′
PBR05_04	4018097	2.32	Avg: 76.49, Stdev: 0.93, Max: 79.2, Min: 74.2	3.31	43.8%	0.92	1.06
PBR05_05	4018097	3.00	Avg: 98.50, Stdev: 0.95, Max: 101.7, Min: 96.0	2.15	44.8%	0.90	1.01
PBR05_12	4018097	2.36	Avg: 77.04, Stdev: 0.71, Max: 79.4, Min: 75.5	2.81	45.5%	0.88	1.04
PBR05_20	4018097	2.38	Avg: 79.43, Stdev: 1.35, Max: 83.6, Min: 75.8	3.31	42.7%	0.96	1.08
PBR05_02	4018356	2.37	Avg: 77.51, Stdev: 0.70, Max: 79.8, Min: 75.5	2.83	45.4%	0.91	1.03
PBR05_03	4018356	2.37	Avg: 77.75, Stdev: 0.84, Max: 81.6, Min: 76.2	3.00	43.8%	0.92	1.07
PBR05_06	4018356	2.91	Avg: 96.46, Stdev: 1.26, Max: 100.8, Min: 92.7	2.39	44.2%	0.94	1.06
PBR05_09	4018356	2.37	Avg: 79.12, Stdev: 1.17, Max: 82.7, Min: 75.8	2.81	44.5%	0.91	1.07
PBR05_13	4025536	4.47	Avg: 149.66, Stdev: 14.13, Max: 184.4, Min: 117.6	1.74	43.9%	0.91	1.10
PBR05_16	4025536	6.48	Avg: 203.97, Stdev: 23.76, Max: 294.1, Min: 157.5	1.96	41.7%	0.89	1.12
PBR05_22	4025536	5.79	Avg: 194.80, Stdev: 16.65, Max: 242.4, Min: 152.4	1.88	41.9%	0.91	1.12
PBR05_26	4025536	4.60	Avg: 157.97, Stdev: 12.91, Max: 182.0, Min: 131.3	2.52	41.2%	0.86	1.16
PBR05_01	4028989	1.94	Avg: 61.68, Stdev: 0.69, Max: 63.5, Min: 59.6	2.68	46.0%	0.90	1.11
PBR05_08	4028989	2.51	Avg: 80.28, Stdev: 0.86, Max: 83.1, Min: 77.3	1.90	46.0%	0.91	1.09
PBR05_15	4028989	2.66	Avg: 86.92, Stdev: 1.10, Max: 90.6, Min: 82.9	1.95	44.9%	0.89	1.03
PBR05_21	4028989	2.50	Avg: 80.86, Stdev: 1.19, Max: 84.4, Min: 76.7	1.95	45.6%	0.90	1.10
PBR05_07	4028990	2.70	Avg: 87.64, Stdev: 1.28, Max: 91.4, Min: 82.7	1.98	44.5%	0.90	1.07
PBR05_11	4028990	2.08	Avg: 66.92, Stdev: 1.02, Max: 70.4, Min: 64.3	2.96	43.0%	0.90	1.13
PBR05_19	4028990	2.07	Avg: 66.19, Stdev: 0.63, Max: 67.9, Min: 64.2	2.78	43.2%	0.92	1.12
PBR05_23	4028990	2.48	Avg: 80.05, Stdev: 1.04, Max: 84.0, Min: 76.8	1.95	44.9%	0.89	1.10
PBR05_10	4029025	2.15	Avg: 67.21, Stdev: 2.39, Max: 73.8, Min: 61.6	3.10	43.7%	0.94	1.04
PBR05_18	4029025	2.39	Avg: 78.84, Stdev: 2.18, Max: 86.4, Min: 74.1	2.46	43.8%	0.90	1.10
PBR05_24	4029025	2.99	Avg: 90.98, Stdev: 2.19, Max: 97.9, Min: 86.0	1.90	44.7%	0.92	1.04
PBR05_27	4029025	2.03	Avg: 65.44, Stdev: 1.22, Max: 69.5, Min: 62.1	2.95	44.0%	0.90	1.09
PBR05_14	4029310	2.82	Avg: 91.16, Stdev: 1.43, Max: 95.7, Min: 86.6	1.92	43.4%	0.91	1.07
PBR05_17	4029310	2.22	Avg: 72.65, Stdev: 0.92, Max: 75.1, Min: 69.8	3.01	42.9%	0.94	1.08
PBR05_25	4029310	2.35	Avg: 77.25, Stdev: 2.48, Max: 83.3, Min: 71.0	2.76	42.7%	0.92	1.05
PBR05_28	4029310	2.20	Avg: 71.88, Stdev: 1.66, Max: 74.9, Min: 67.4	2.85	42.4%	0.95	1.08

These findings were confirmed by two individual non-pooled samples, each run twice (Table 4). For more details on these QC parameters, please refer to Affymetrix notes on U133Plus2 microarrays.

**Table 4:** Non-pooled samples replicates.

Sample ID	Lot #	RAW Q	Background	SF	Present	GAPDH 3′/5′	Actin 3′/5′
N23C-1	4025534	7.02	Avg: 258.22, Stdev: 50.78, Max: 384.7, Min: 139.5	3.10	35.6%	0.93	1.11
N23C-2	4025534	5.62	Avg: 192.50, Stdev: 13.21, Max: 220.6, Min: 164.5	2.44	39.0%	0.87	1.10
N82B-03	4025534	2.75	Avg: 83.76, Stdev: 5.67, Max: 96.5, Min: 73.5	2.62	43.2%	0.86	1.15
N82B-04	4025534	4.97	Avg: 164.27, Stdev: 14.91, Max: 212.6, Min: 126.8	2.96	39.8%	0.87	1.13

For more details on these QC parameters, please refer to Affymetrix notes on U133Plus2 microarrays.

**Figure 1:**
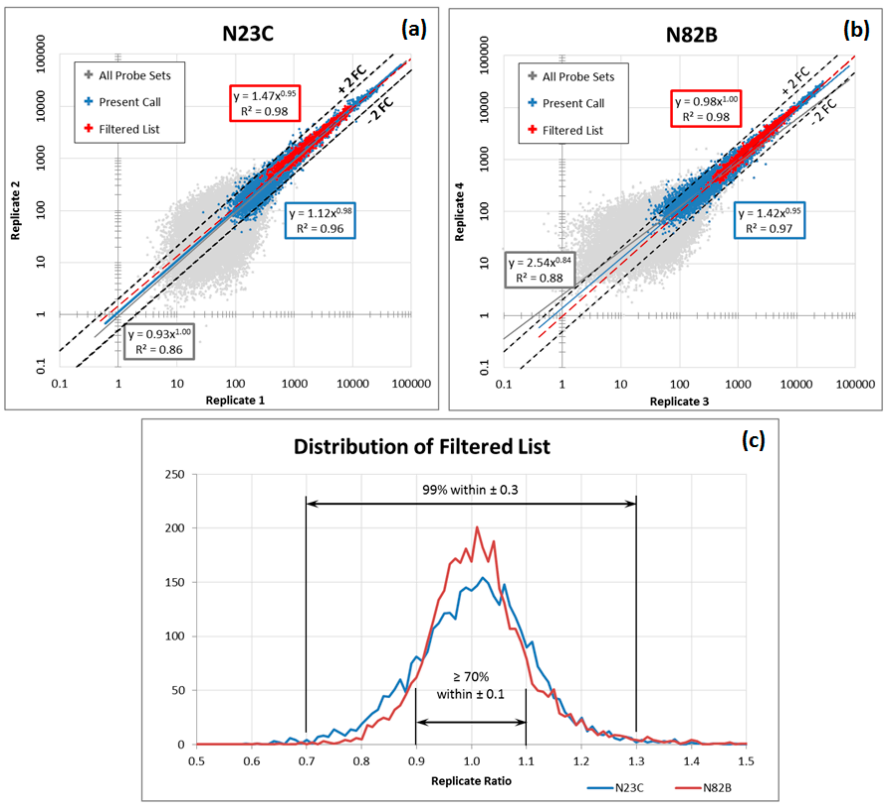
Technical Replicate Hybridization. (**a**,**b**) Correlation between two replicates hybridizations of samples N23C and N82B; (**c**) distribution of replicate ratios for probe sets within the “filtered” list.

Probe sets with expression levels lower than 100 were classified as too “noisy” based on the data from these repeated hybridizations, whereas those with expression levels greater than 10,000 were classified as “saturated” and unreliable for detecting change in expression (Figure 1). The probe sets must also belong on the validated list published by the MAQC study as well as verified to be repeatable on our own EDTA and PAXgene replicate experiments. Finally, any outliers are also excluded because of their uncharacteristic expression value. These steps are summarized in Table 5.

**Table 5:** Probe Set Filtering.

Present in All Samples
Expression between 100 and 10,000
On MAQC list
On EDTA stable list (±10%)
On PAXgene stable list (±15%)
Outlier: >2-fold outside of 95%-tile range

#### 2.3.3. Pairs of Genes

**Figure 2:**
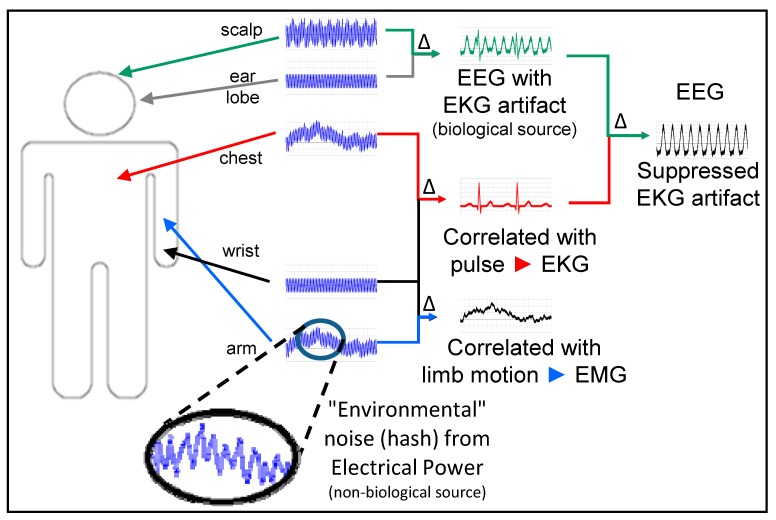
EKG pair is “suppressor” used with “raw” EEG pair to obtain a clean EEG signal with reduced EKG artefacts. Specifically selected raw signals which seem to be useless noise, are combined to suppress masking noise, revealing the underlying useful information that was always present.

Pairs of genes are the minimum unit for analysis when using self-normalization to suppress confounding factors, such as the diurnal cycle [22]. From the stable probe sets identified in the previous steps, pairs of genes were evaluated as ratios. A pair with an AUROC value of 0.7 or higher was classified as “significant”, and the pair was set aside as a candidate biomarker pair for further combination analysis. This AUROC value was selected from empirical experience as a good balance between potentially excluding valid probe sets and accepting those that would eventually prove to be unreliable in further evaluations. Pairs using the sum of the gene expression were also set aside as candidates if the pair had complementary noise which was reduced by the sum, as evidenced by a significant increase in AUROC above that of the individual genes. Additionally, if a pair had little correlation with the disease under study (AUROC~0.5), but showed good correlation with the significant pairs, then the pair was also set aside as a potential “suppressor” pair [23]. Finally, combinations of candidate biomarker and suppressor pairs were evaluated by AUROC and a short list was selected for validation on a test set or by multiple iterations of n-fold cross-validation. The concept of using pairs of genes is similar to the practice of using differential signals in electrocardiogram (EKG) and electroencephalogram (EEG) measurements (Figure 2). The desired signal is obscured by the electrical noise from both the external environment and spurious muscle contractions in the body of the patient. However, by selecting appropriate reference points to obtain a “suppressor” signal, it is possible to optimally recover the desired signal.

#### 2.3.4. One-Against-All (Orthogonality)

One of the difficulties of using whole blood is that its transcriptome contains information reflecting not only many diseases but also all kinds of other confounding factors. However, this breadth of information is also the key to the solution of the problem. Since so many factors are mirrored in the blood transcriptome, it should be possible to find a signature for each, and reduce the problem to a set of “independent” or orthogonal equations for which the solution becomes nearly trivial.

This solution is based on our hypothesis that, whereas many genes are affected by more than one disease or condition, there may exist combinations of genes that are affected only by a single condition. By setting up an analysis to look for only those combinations of genes that respond to a single condition in the explicit presence of confounding factors such as other diseases, we will be able to identify those genes that match the independent equation case. One beneficial side effect of this solution is that sample acquisition becomes much simpler; it is not necessary to find samples from patients with two or more diseases or conditions of interest. The practical consequence is not trivial: for diseases with very low prevalence rates, patients with multiple disease combinations would be vanishingly rare and impossible to acquire.

We assign the “other” samples to the “not-this-disease” group and take advantage of the relatively larger numbers to attenuate any “out of the ordinary” characteristics of an individual sample. As the number increases, the potential skew from any one individual is diluted. Additionally, the gene panel is trained to reject the signature of all other diseases included in the “other” samples. This is the “one-against-all” approach for analysing gene expression profiles that makes each prediction panel more specific to the target disease condition.

#### 2.3.5. Group Balance

A side effect of employing the one-against-all approach is that clinical information about a patient in a research study is usually limited to the condition being studied. For instance, in a colorectal cancer study, all the patients under study will be endoscopically examined and determined to either have colorectal cancer (case) or to be free from colorectal cancer (control). However, it is not usually possible to know for certain whether patients from other studies are truly free from colorectal cancer. Researchers can only assume that it would be unlikely for these patients to have colorectal cancer, which is a disease with a low prevalence (<1%). The problem is that there are many more of these patients with unconfirmed diagnoses as compared with the colonoscopy-verified cancer-free patients. It might be possible to incorrectly predict all confirmed colorectal cancer negative patients as false positive and still achieve a very high specificity by correctly predicting the samples from other studies as colorectal cancer-free. To account for such bias, statisticians weigh more heavily the relative contribution of high-quality data (verified pathology) relative to the larger amount of low-quality data (assumed colorectal cancer-negative pathology).

In our approach, this accounting for bias is achieved by replicating the samples that need to be weighted more heavily. We chose replication because it has the added benefit that we can introduce a controlled amount of random Gaussian noise to simulate the effect of measurement uncertainty and reduce the impact of any single data point that might skew the results. To increase the contribution of the confirmed non-cancer cases, we replicated each PAXgene HCC sample 15 times and each EDTA HCC sample 20 times, to balance them with each other and with the samples from other studies. (Table 6).

**Table 6:** Training Set Group Balance.

Group	Training Set			Replicates/Sample		Combined					
	PAX	EDTA	Sub Total	PAX Repl.	EDTA Repl.	PAX Eff.		EDTA Eff.		Sub Total	
HCC	26	20	46	15	20	416	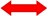	420		836	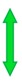
HpB	28	0	28	0	0	28	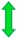	0	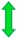	38	
Control	28	7	35	14	56	420	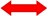	399		819	
Other	0	830	830			0		830		830	
Total	82	857	939			864		1649		2513	

The two horizontal red arrows indicate the balance between PAXgene and EDTA collected data, the in-between vertical green arrow indicates the balance between HCC and Control subgroups. The two vertical green arrows indicate the balance between Cancer and Control/Other subgroups

#### 2.3.6. Search Speed Optimization

However, the solution proposed above has the consequence of increasing both the size of the data set and the number of potential combinations to be evaluated to the point that it becomes too time-consuming to conduct a systematic search. Since the goal is only to find some combinations of genes that predict disease well enough to be useful, we used a Monte-Carlo approach to accelerate the search.

**Figure 3:**
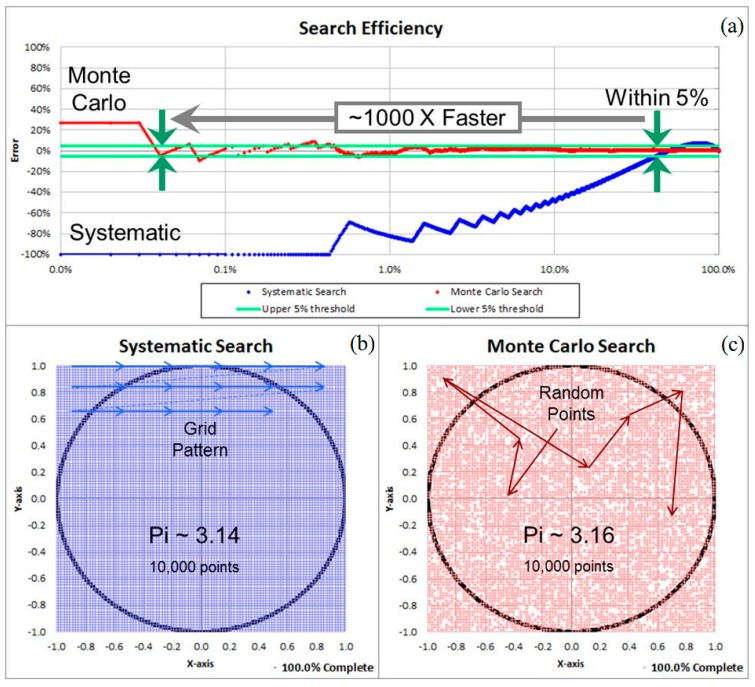
Estimating the value of π numerically. (**a**) Convergence speed using a systematic search (**b**) and a Monte-Carlo random search (**c**).

The efficiency of a random Monte-Carlo evaluation can be illustrated by comparing the numerical estimation of the value of π with and without Monte-Carlo acceleration. The mathematical constant, π, represents the ratio of the area within a circle to the square of its radius. For a circle of unit radius with an enclosed area of π units squared, the enclosing square has sides of 2 units with an area of 4 units squared. A systematic evaluation would divide the enclosing square into a grid with regularly spaced points and determine whether each of these points is within the circle or outside it. The ratio of the number of points within the circle to the number of points inside the square is an approximation of π/4. A random search would select points at random rather than systematically march along the grid. As illustrated in Figure 3, the Monte-Carlo estimate comes within 5% of the correct value much more rapidly than the systematic evaluation.

#### 2.3.7. New Samples and Cross-Validation

The method was applied to a group of blood samples from ten different diseases and controls. We used the entire data set of ten diseases and controls to identify gene expression biomarkers associated with each of the ten diseases using the statistical method described above (one-against-all). This is the first data set collected entirely in PAXgene tubes. Because the sample numbers are still small, it is not practical to evaluate prediction performance by partition into a traditional training/test sets. Instead, we performed a 2-fold cross-validation iterated 1000 times.

## 3. Results and Discussion

We applied these procedures to a set of samples that had the complication that the samples had been collected using two different technologies: EDTA and PAXgene tubes. Although the raw expression levels exhibited differences between these two sets, using our strategy it was possible to find combinations of pairs that exhibited improved stability and were predictive of the underlying disease.

**Figure 4:**
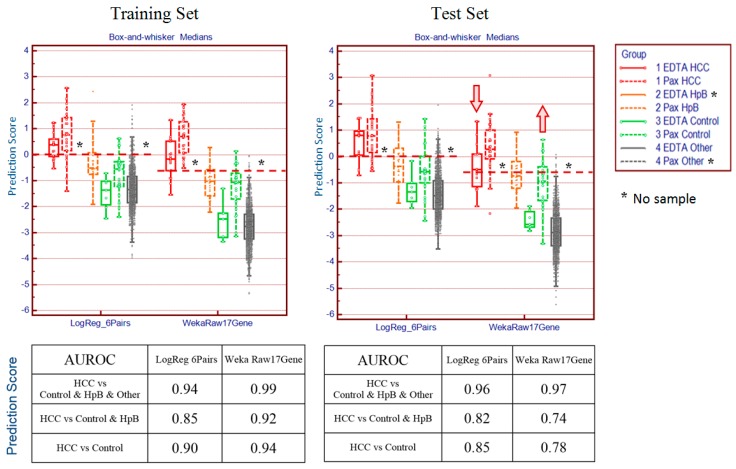
Prediction scores using the method described in this paper (LogReg_6Pairs) Weka prediction using all data without any preprocessing (Weka Raw17Gene).

The search identified a combination of 12 probe sets in six pairs from the filtered list of 586 candidates. This combination scored the samples with fairly good overlap between the PAXgene and EDTA samples in each disease category in both the training and independent test sets (Figure 4).

For comparison we also plotted the predictions using a standard open-source statistical package, Weka. This analysis was conducted using the SimpleLogistic classifier function with default parameters employing all available 9163 probe set data without any filtering or weighting. The SimpleLogistic classifier has a built-in feature selection capability that selected the final set of 17 probe sets from the entire data (WekaRaw17Gene). This comparison using an unmodified version of Weka under default settings is not intended to critique Weka, but rather to highlight the effect of the additional steps described in this manuscript.

The test set results from the WekaRaw17Gene panel trained without sample weighting show that the liver cancer samples collected in EDTA tubes (median = −0.50) have dropped in prediction scores relative to the PAXgene cancer samples (median = +0.28) and are now in the same range as chronic hepatitis B (median = −0.74) and control patients (median = −0.94). By contrast, the predictions using our method aligns both liver cancer groups (EDTA median = +0.80, PAXgene median = +0.77) which are well separated from the HpB samples (median = −0.38) and control samples (EDTA median = −1.34, PAXgene median = −0.57).

These results illustrate that the novel statistical method described above worked well in separating the three groups of liver cancer study samples (HCC, HpB, control), even under different conditions, such as chip lot or blood collection tubes. Additionally, the loss of test set prediction accuracy resulting from the absence of the PAXgene HpB and other subgroups of samples can be overcome by using the information from other available PAXgene subgroups, as shown in the results presented in Figure 4.

**Figure 5:**
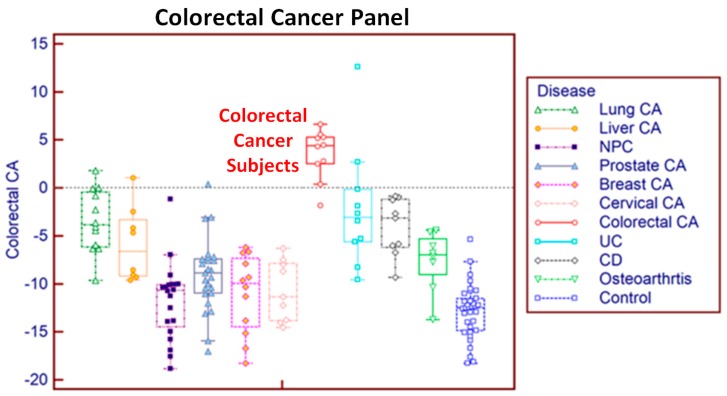
Prediction of risk for colorectal cancer for individual subjects using the colorectal cancer gene pair panel identified by the method described in this paper.

**Figure 6:**
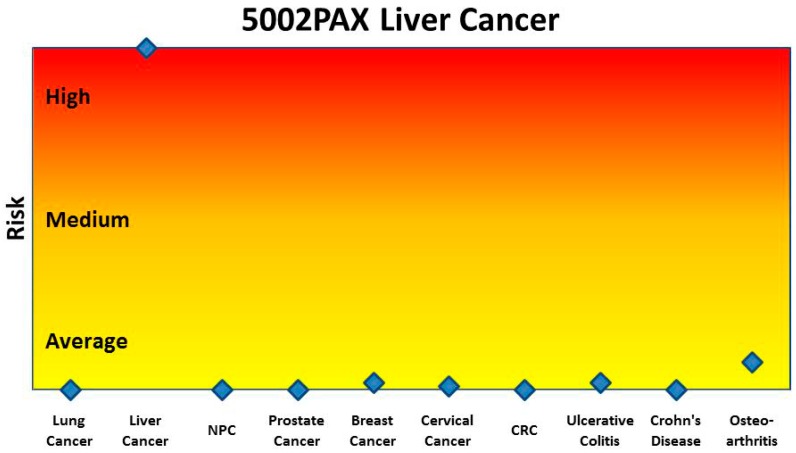
Prediction of the risk of 10 different diseases for an individual liver cancer patient, using the gene pair panels obtained using the method described in this paper. This patient was known to have liver cancer and had no indication of any of the other diseases being evaluated.

We then applied the same procedures to a group of blood samples from ten different diseases and controls collected in PAXgene tubes (Table 2). The results of 1000 iterations of 2-fold cross-validation for each disease’s gene pair panel are summarised in Table 7. When this method was used, all ten disease gene pair panels showed consistently high prediction performance: high sensitivity (mean 89%), high specificity for the healthy controls (mean 98%), high specificity for the other nine diseases (93%), and high AUROC (mean 96%). The prediction results for each individual subject for the risk of colorectal cancer are charted in Figure 5, which shows good discrimination between colorectal cancer and controls and the other nine diseases. All but one colorectal cancer subject achieved a score above the threshold value of 0 while only four of all the other subjects returned a false positive prediction. The other gene panels achieved similar results. Figure 6 is an example of the prediction of the risk of ten different diseases for a single liver cancer patient using the gene pair panels obtained using the method described in this paper. This liver cancer patient can be seen to be at high risk for liver cancer and at no higher than population average risk for the other nine diseases.

**Table 7:** Performance of gene pair panels of each disease using our statistical method (1000 iterations of 2-fold cross validation).

Disease	Sensitivity	Control Specificity	Others Specificity	AUROC
Lung Cancer	100%	100%	91%	95.7%
Liver Cancer	100%	100%	95%	99.5%
Nasopharyngeal Cancer	75%	100%	94%	95.1%
Prostate Cancer	76%	91%	80%	82.9%
Breast Cancer	100%	100%	95%	99.5%
Cervical Cancer	89%	100%	94%	96.7%
Colorectal Cancer	90%	100%	95%	98.3%
Ulcerative Colitis	70%	94%	92%	89.8%
Crohn’s Disease	100%	100%	94%	99.3%
Osteoarthritis	100%	97%	98%	99.4%

## 4. Conclusions

We have presented a procedure that identifies a set of probe sets which demonstrate reliable expression levels for target genes. Using these, we evaluated ratio pairs to achieve self-normalization. By combining discriminative pairs and suppressor pairs, we found useful panels of gene pairs that are able to predict disease even across varying conditions such as chip lot or sample collection tube differences.

**Figure 7:**
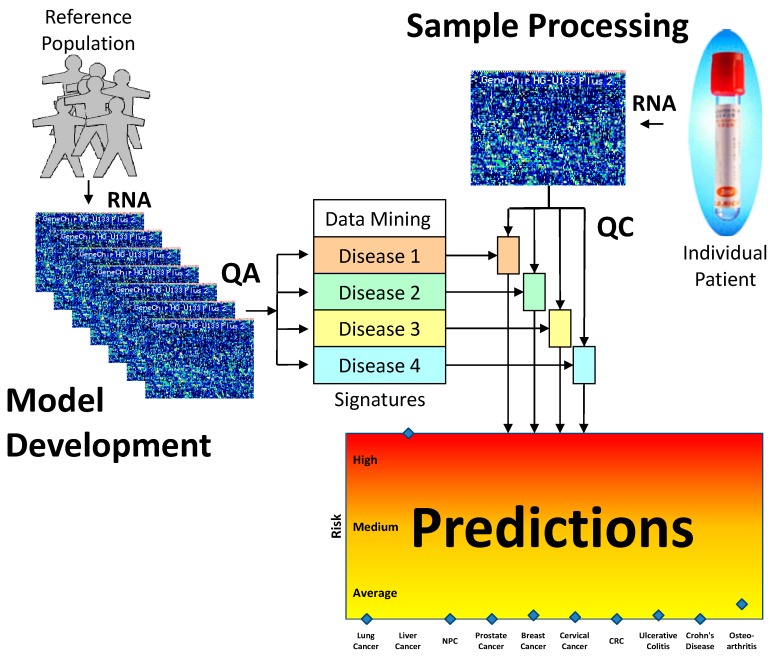
Schematic representation multiple disease prediction. The gene expression from a reference population representing several disease conditions is filtered according to a Quality Assurance system based on repeatability data. These data are then analysed to derive predictive model for each disease condition. These models can then be applied to the data from a new sample to make risk prediction for this individual.

For comparison, we also processed the data with a widely-used machine learning package, Weka, using the SimpleLogistic model with automatic feature selection. Weka achieved the best overall discrimination with a panel of 17 probe sets, but was unable to suppress the bias introduced by the use of two different collection tubes. That is, whereas our method managed to align the liver cancer samples so that the majority (~75%) of both EDTA and PAXgene samples are predicted as true positive in the test set, the Weka predictions (based on data using unfiltered gene lists and without sample weighting) classified nearly half the liver cancer samples collected in EDTA tubes as false negatives and nearly all of the liver cancer samples as true positives (Figure 4). Our method was then applied to a new cohort of samples collected in PAXgene tubes representing ten different diseases. The panels predicted with consistently high performance under repeated 2-fold cross-validation. We expect, based on our previous experience with EDTA data that the prediction performance will hold when the sample number is increased. It may even be possible that with increased sample numbers, the gene panels may be refined and result in improved prediction performance. With this method, the risk of a single patient having any of the ten diseases studied can be obtained simultaneously using a single blood sample. Figure 7 is a schematic representation of the process by which we identify panels of genes that are predictive of disease conditions. These panels can then be applied to the data from a single individual to make predictions of risk for these conditions.

We present the methodology described in this paper as a minimum set of procedures optimized for noisy data with individual component performance complementary to the other components. For example, the use of seemingly non-informative genes is suggested by the fundamental concept of suppressor variables, which dates back to 1941 and is similar to the differential amplifier of 1934 [24] or even the Wheatstone bridge of 1843 [25] and has been successfully applied in other areas of science and technology but appears to have been generally neglected by the community of scientists involved in genomic data analysis. We are convinced that a return to a more balanced holistic approach to data analysis may help in extracting useful information from the mass of data which can be obtained by rapidly advancing modern technology.

The circulating peripheral blood system is involved in the regulation, coordination, metabolism and immune maintenance of all cells, tissues and organs. Functions of blood include transporting nutrients, oxygen and biomolecules, and removing cellular waste. Blood is further involved in immune surveillance throughout the body, and delivery of immune factors and mediators to sites of disease, infection and injury. Thus, the circulation and physiologically interactive nature of blood ensure that this system encounters, transmits, and is affected by a wide range of biological signals.

Over the past decade, we have investigated the blood genetic signatures of a wide variety of diseases and conditions affecting numerous organs and functions, including psychiatric disorders, osteoarthritis, cardiovascular disease, gastrointestinal diseases, and cancer. It has been found that each disease has its characteristic expression spectrum of genetic signatures in peripheral blood, which make it possible to detect disease anywhere in the body by accessing subtle changes in blood RNA. Based on these studies, we address the Sentinel Principle^®^ that views the circulating blood cells as “sentinels” for detecting and responding to micro-environmental changes in the body. Accordingly, the current state of health or disease of an organism is conveyed in the blood through interactions between circulating blood cells and the body’s cells, tissues and organs. Since blood samples can be readily obtained non-invasively, the genetic signature derived from blood RNA provides an alternative to tissue biopsy for determining the diagnosis and prognosis of many different diseases. Overcoming the problems of blood-based transcriptomics discussed above will further extend the application of the Sentinel Principle not only to the diagnosis of multiple diseases in one blood sample, but also to other fields of personalized medicine such as active surveillance, prognosis, drug response, and so on.

## Abbreviations

area under the receiving operator characteristic curve (AUROC); electrocardiogram (EKG); electroencephalogram (EEG); hepatocellular cancer (HCC); chronic hepatitis B (HpB).
